# Hypoxia exposure and B‐type natriuretic peptide release from Langendorff heart of rats

**DOI:** 10.1111/apha.12767

**Published:** 2016-08-26

**Authors:** K. Anttila, T. Streng, J. Pispa, M. Vainio, M. Nikinmaa

**Affiliations:** ^1^ Laboratory of Animal PhysiologyDepartment of BiologyUniversity of TurkuTurkuFinland; ^2^ Department of Pharmacology, Drug Development and TherapeuticsUniversity of TurkuTurkuFinland; ^3^ Turku Center for Disease Modeling (TCDM)University of TurkuTurkuFinland

**Keywords:** B‐type natriuretic peptide, hypoxia, Langendorff heart, oxygen consumption

## Abstract

**Aim:**

We studied whether available oxygen without induced mechanical stretch regulates the release of the biologically active B‐type natriuretic peptide (BNP) from Langendorff heart.

**Methods:**

Rat hearts were isolated and perfused with a physiological Krebs–Henseleit solution at a constant hydrostatic pressure in Langendorff set‐up. The basal O_2_ level of perfusate (24.4 ± 0.04 mg L^−1^) was gradually lowered to 3.0 ± 0.01 mg L^−1^ over 20 min using N_2_ gas (*n *=* *7). BNP and O_2_ level were measured from coronary flow. During control perfusions (*n *=* *5), the O_2_ concentration was kept at 26.6 ± 0.3 mg L^−1^.

**Results:**

A low oxygen concentration in the perfusate was associated with a significant increase in BNP release (*F* = 40.4, *P *<* *0.001). Heart rate decreased when the oxygen concentration in the perfusate reached 9.1 ± 0.02 mg L^−1^ and continued to fall in lower oxygen concentrations (*F* = 14.8*, P *<* *0.001). There was also a significant but inverse correlation between BNP and oxygen in the coronary flow (*R* ^2^ = 0.27, *P *<* *0.001).

**Conclusion:**

In the spontaneously beating Langendorff rat heart, a decreasing concentration of oxygen in the ingoing perfusion increased the secretion of BNP. The effect of oxygen was independent of mechanical stretch of the heart as it occurred even when the heart rate decreased but the pressure conditions remained constant. The difference in the oxygen capacitance of blood and Krebs–Henseleit solution appears to be a major factor affecting secretion of BNP, which is correlated with the oxygen tension of myocardial cells and affected both by the oxygen concentration and capacitance of solution perfusing the heart and by the coronary flow.

Lang *et al*. (1985) showed that a large and rapid intravascular volume increase elevated the plasma levels of A‐type natriuretic peptide (ANP) in the isolated heart of rat. This finding was the starting point of many studies, which have resulted in the currently accepted conclusion that the primary stimulus for the natriuretic peptide release, including the B‐type natriuretic peptide (BNP), is either mechanical stretch or pressure alone (Ruskoaho 1992, Tokola *et al*. 2001). However, hypoxia has also proved to be a powerful stimulus for the release of natriuretic peptides from the rodent heart (Baertschi *et al*. 1986, Lew & Baertschi 1989, Uusimaa *et al*. 1992a,b, Chen *et al*. 1993, Arad *et al*. 1994, Tóth *et al*. 1994, Focaccio *et al*. 1995, Svorak *et al*. 1996, Zhang *et al*. 2004) and from isolated cell lines of human cardiac origin (Ljusegren & Andersson 1994, Klinger *et al*. 2001, Hopkins *et al*. 2004, Casals *et al*. 2009). Also, both the A‐ and B‐type peptides have a hypoxia‐responsive element in the promoter sequence of their respective genes (Chun *et al*. 2003, Luo *et al*. 2006) and hypoxia has been shown to be a direct and sufficient stimulus for the transcription of the gene encoding B‐type peptide (Weidemann *et al*. 2008). The possible role of hypoxia in natriuretic peptide function has been reviewed, for example, by Arjamaa & Nikinmaa (2009).

Mechanical stress/pressure and hypoxia as stimuli causing natriuretic peptide secretion may actually be closely related. All the changes in the mechanical work of heart cells are associated with changes in the oxygen consumption of the cells (Loiselle *et al*. 2008). Consequently, a mechanical stretch, known to cause natriuretic peptide release, causes hypoxia. It is possible that what is ascribed to the mechanical stress/pressure effects are actually due to their influence on oxygen equilibria, as a large effect is observed in *in vitro* preparation (in the absence of blood cells) but not *in vivo* (Lang *et al*. 1985, Sakata *et al*. 1988). Because of the 1/30th oxygen capacity of Krebs–Henseleit solution (regularly used as perfusing solution in Langendorff set‐up) as compared to native blood, an acute volume expansion will cause a marked reduction in the oxygen tension of myocardial cells, with the prediction that a large amount of natriuretic peptide in the Krebs–Henseleit‐perfused hearts is released, but only small changes are seen in intact animals as a result of increased oxygen consumption of the cells.

In the work presented here, we have further investigated whether hypoxia as such can cause the release of B‐type natriuretic peptide in Langendorff preparation in the absence of pressure changes. Previously, Lew & Baertschi (1989) have presented data that this is the case for ANP. In addition to regulating and measuring the ingoing oxygen concentration, we have determined it after the flow of Krebs–Henseleit solution through the spontaneously beating rat heart.

## Materials and methods

### Animals

Twelve (five control and seven hypoxia‐treated) hearts from adult male Sprague Dawley rats (Harlan Laboratories, the Netherlands) were used in the study. Before the experiments, the rats were maintained at the Central Animal Laboratory, University of Turku, Finland, on a 12‐h light/dark cycle with free access to bottled tap water and pelleted rat chow (CRM, Witham, Essex, UK). The National Animal Experiment Board approved the study protocol. All experiments were conducted in accordance with the relevant legislation and the guidelines of the International Council for Laboratory Animal Science.

### Experimental set‐up

The hearts were removed during anaesthesia (i.p. choral hydrate, 900 mg kg^−1^), and heparin (3000 IU) was injected i.v. to prevent blood clotting. After removal of the heart, the ascending aorta was connected into Langendorff heart perfusion apparatus (Radnoti Working Heart System, Monrovia, CA, USA) and was subjected to retrograde perfusion with a physiological Krebs–Henseleit solution (NaCl 115 mm, KCl 4.6 mm, KH_2_PO_4_ 1.2 mm, MgSO_4_ 1.2 mm, CaCl_2_ 2.5 mm, NaHCO_3_ 25 mm and glucose 11 mm) equilibrated with carbogen gas (95% oxygen and 5% carbon dioxide) to a pH of 7.4. Special care was taken that aorta was cannulated correctly (i.e. aortic valve was not penetrated). Thus, all the perfusion solution went through coronary circulation only.

**Figure 1:**
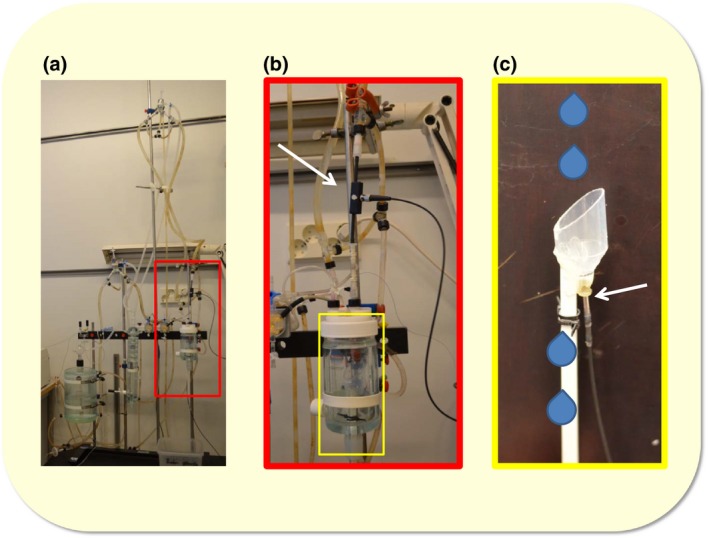
Langendorff measurement set‐up. (a) Whole system, (b) enlargement from a (red rectangle) showing details of the oxygen level measurements before the heart (an arrow shows the probe) and heart chamber and (c) enlargement from b (yellow rectangle) showing the chamber that was used to measure the oxygen level of coronary flow (an arrow shows the probe) and collect samples from the flow. Coronary flow was dripping to the chamber equipped with the oxygen probe, and after recording the oxygen level, the coronary flow entered into a collecting tube, which made it possible to analyse samples from the coronary flow.

Perfusion was initiated (peristaltic pump, Masterflex, 7518‐00, Cole‐Parmer Instruments Company, Vernon Hills, IL, USA), and a constant hydrostatic pressure was introduced in a non‐recirculating manner with a constant basal perfusion pressure of 50–60 mmHg. The Langendorff apparatus was equipped with oxygen electrodes (OXROB10 optical oxygen probe connected to a FireSting O_2_ meter; PyroScience GmbH, Aachen, Germany) to measure the oxygen concentration in the perfusion solution before it entered the heart (Fig. 1a,b), and from coronary flow (Fig. 1c). Afterload pressure, heart rate and organ temperature measurements were recorded during hypoxia exposure. The basal O_2_ level (24.4 ±0.04 mg L^−1^) was gradually lowered to 3.0 ±0.01 mg L^−1^ using N_2_ gas within 20 min (the exact time depending on the heart coronary flow).

Both the perfusion solution and the heart were maintained at 38 °C. The left ventricular pressure was measured through a lateral connection from the perfusion cannula with a pressure transducer (SP 844, Memscap AS, Durham, NC, USA) connected to the data acquisition system (ML870, PowerLab 8/30, ADInstruments, Oxford, UK). Left ventricular pressure (systolic and diastolic) was recorded, and the heart rate was obtained from the pressure curve. These hemodynamic parameters were recorded on a personal computer using Chart for windows ver. 5 software and powerlab data acquisition system for acquiring and analysing the data. The experimental set‐up is detailed in Figure 1.

### BNP analyses

Coronary flow samples were collected at specific time points (Fig. 1c) representing certain oxygen levels of the perfusate as shown in Table 1. The samples were stored at −80 °C (for 1–5 weeks). BNP_1‐32_ was measured with an enzyme immunoassay (EIA; Cat no FEK‐011‐14, Phoenix Pharmaceuticals, Burlingame, CA, USA). Briefly, a 50 *μ*L sample aliquot was bound to a BNP antibody and competed with a biotinylated peptide. Biotinylated peptide interacts with streptavidin–horseradish peroxidase which catalyses the reaction with the fluorescent substrate. Fluorescence was measured with an Enspire 2300 Multilabel Reader (PerkinElmer, Turku, Finland). Wavelength for excitation was 325 nm and for emission 420 nm.

**Table 1:** Time points for the collection of BNP samples and the corresponding oxygen levels in the perfusate. Hypoxia was produced with N_2_ gas, and the oxygen concentration was measured with an optical oxygen probe. The values are mean ± SE

Time point (min)	Oxygen level in hypoxia exposure (mg L^−1^)	Oxygen level in controls (mg L^−1^)
0	24.4 ± 0.04	27.1 ± 0.7
4.5	15.2 ± 0.05	26.9 ± 0.8
5.0	13.1 ± 0.02	26.8 ± 0.8
7.5	10.06 ± 0.01	26.8 ± 0.8
8.5	9.08 ± 0.02	26.7 ± 0.9
9.5	8.04 ± 0.01	26.6 ± 0.9
10.5	7.04 ± 0.01	26.5 ± 0.9
12.0	6.03 ± 0.003	26.5 ± 0.9
13.5	5.03 ± 0.01	26.4 ± 0.9
19.5	3.01 ± 0.01	26.2 ± 0.8

### Statistical analysis

Statistical analyses were performed using sigmaplot 12.3 (Systat Software, San Jose, CA, USA). A significance level of *α* = 0.05 was used in all statistical tests. Data normality and homogeneity were tested with Kolmogorov–Smirnov and Levene tests respectively. A general linear model of two‐way repeated‐measures anova was used to analyse the BNP level, removal of oxygen ((oxygen concentration in Krebs–Henseleit solution entering heart – oxygen level in coronary flow)/(oxygen concentration in Krebs–Henseleit solution entering heart)*100) and heart rate variability between experimental groups and oxygen concentrations in the perfusate, after which *a posteriori* Holm–Sidak test was performed. A correlation between the oxygen concentration in coronary flow and its BNP level was evaluated with linear regression.

## Results

**Figure 2:**
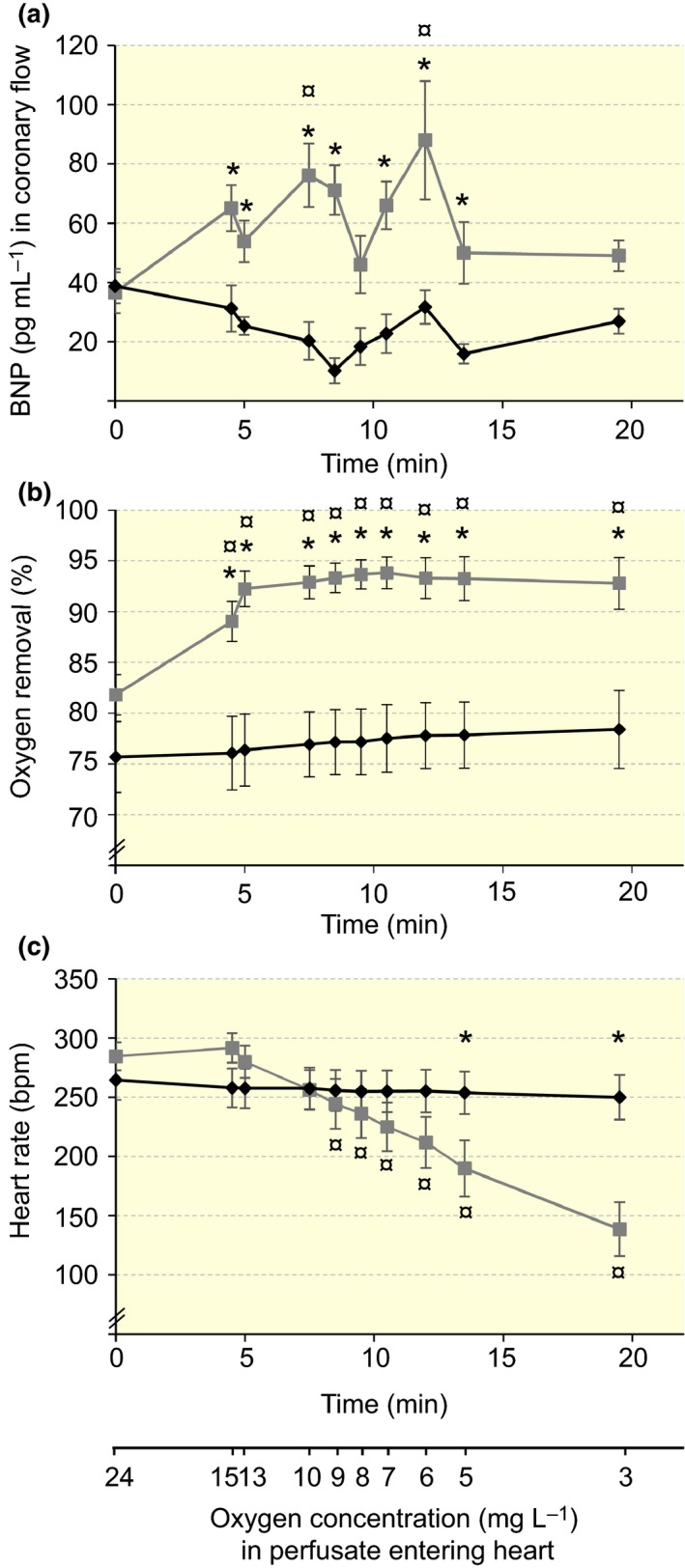
(a) BNP concentration in coronary flow, (b) oxygen removal from perfusate and (c) heart rate in the Langendorff rat heart when the oxygen concentration was decreased with N_2_ gassing (hypoxia group; 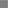, *n* = 7), controls (♦, *n* = 5). Oxygen was measured with an optical oxygen probe before and after the heart during perfusion. *Denotes a significant difference (*P *<* *0.05) between hypoxia treatment and controls, while ¤ indicates a significant difference (*P *<* *0.05) within a hypoxia group as compared to the starting value. Two‐way repeated‐measures anova followed by Holm–Sidak test was used. The values are mean ± SE.

**Figure 3:**
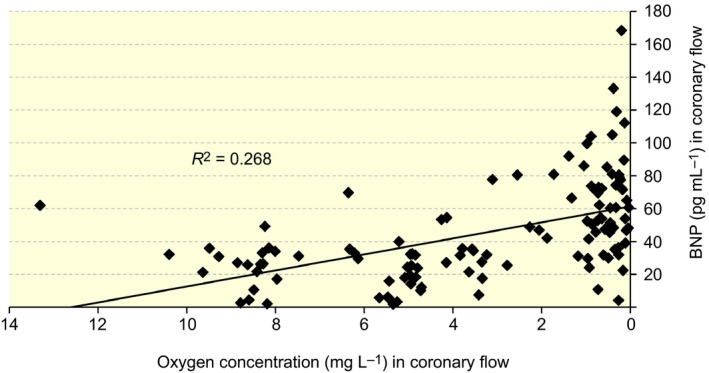
The correlation between the oxygen concentration and BNP level in coronary flow.

A significant interaction between main factors, that is experimental groups and time after the start of the exposure (oxygen level in perfusate), was observed in the BNP level (*F*_1,9_ = 2.3, *P* = 0.022; Fig. 2a). In controls, the BNP level remained at 24.3 ± 1.9 pg mL^−1^. However, there was a significant increase in the BNP during hypoxia exposure at 7.5 min (76.2 ± 10.8 pg mL^−1^) and 12 min (88.0 ± 20.0 pg mL^−1^) after starting the treatment, representing oxygen concentrations of 10.1 ± 0.01 and 6.0 ± 0.003 mg L^−1^, in perfusate entering the heart (*T* < 3.5, *P *<* *0.03). Furthermore, there were significant differences between control and hypoxia‐treated hearts (*F*_1_ = 40.4, *P *<* *0.001) during the experiment except at the oxygen concentrations of 24.4 ± 0.04, 8.0 ± 0.01 and 3.0 ± 0.01 mg L^−1^. The BNP level also correlated significantly with oxygen level in coronary flow (*R* ^2^ = 0.27, *P *<* *0.001); the BNP concentration increased when the oxygen concentration in the coronary flow decreased (Fig. 3).

The oxygen removal data indicates how little oxygen the Krebs–Henseleit solution actually carries. Although the perfusate entering the heart had oxygen concentration over five times of that in native blood, the heart consumed 77.1 ± 1.0% of this oxygen in the controls (i.e. contained less than 25% of the oxygen entering the heart), while the venous blood normally has 60–70% of the oxygen in the arteries. When the oxygen concentration entering the heart was decreased, the removal of oxygen increased in 5 min from the initial 81.8 ± 2.0 to 93.2 ± 0.6% [*F*_9_ = 15.5, *P* < 0.001 time as factor (Fig. 2b)]. The 5‐min time point corresponds to the oxygen concentration of 13.1 ± 0.02 mg L^−1^ (a concentration still higher than that of arterial blood). Thereafter, virtually all the oxygen entering the heart was used up; that is, the removal of oxygen (in %) remained high and constant. The reason for the difference between the perfusing solution and blood is due to their markedly different oxygen capacitances, that is the molar amount of oxygen needed to cause a unit change in measured dissolved oxygen concentration (or partial pressure of oxygen).

There was a significant interaction between experimental groups and treatment (*F*_1,9_ = 11.4, *P *>* *0.001) when the heart rate was analysed (Fig. 2c). In hypoxia, the heart rate started to decrease at 8.5 min, that is when the oxygen concentration had decreased to 9.1 ± 0.02 mg L^−1^ (*F*_9_ = 14.8, *P *<* *0.001 for time as factor). Thereafter, there were also significant differences in the heart rate between control and hypoxia exposure at 13.5 and 19.5 min; the corresponding oxygen levels were 5.0 ± 0.01 and 3.0 ±0.01 mg L^−1^ (*T* < 2.3, *P *<* *0.035). In controls, both the oxygen concentration (26.6 ± 0.3 mg L^−1^) and the heart rate (256 ± 5 bpm) remained constant during the experiment.

## Discussion

Previously, Lew & Baertschi (1989) have shown that the ANP release from Langendorff preparation is increased by hypoxia. In our study, we showed that this is also the case for the release of BNP from an isolated heart perfused with a clear buffer solution. It is important that the release of both the A‐type and B‐type natriuretic peptides has now been shown to respond to hypoxia, as ANP is released mainly from atria (Ruskoaho 1992), while the major site of BNP expression is the ventricle (de Bold *et al*. 1996). In the present study, the perfused isolated rat heart was exposed to constant mechanical load and was spontaneously beating. When the ingoing oxygen concentration was decreased in the perfusion system, the BNP secretion started to increase, and at the highest the concentration was about threefold as compared to the control level. Notably, the BNP secretion was still elevated when the heart rate started to decrease. Both these findings indicate that the mechanical work of the heart does not need to increase for increased BNP release to take place. Indeed, it has earlier been shown that hypoxia without mechanical work causes natriuretic peptide release. For example, hypoxia without myofilament movement caused the release of natriuretic peptide from the myocardial cells of salmon (Arjamaa *et al*. 2014). Furthermore, hypoxia triggered BNP expression in isolated human atria independent from any mechanical stress (Möllmann *et al*. 2010).

However, in the current study, when hypoxia became deeper (i.e. oxygen concentration of perfusate decreased below 5 mg L^−1^), the BNP concentration in the perfusate decreased. This occurred simultaneously with the decrease in heart rate and flow of perfusate, which indicates that the heart was fatigued. A similar situation has been found in a modified Langendorff perfusion system in which the ventricular myocardium was mechanically stretched with a constant load. The release of both A‐type (Kinnunen *et al*. 1992) and B‐type (Pemberton *et al*. 2005) peptides decreased after the initial increase caused by the mechanical stretch in spite of a constant stimulus. Again, to us this indicates that excretion cannot continue in fatigued heart (in which the oxygen tension gradient is much diminished and oxygen consumption of the cells decreased).

These results, and also the findings that whereas a marked natriuretic peptide secretion occurs in Krebs–Henseleit solution‐perfused Langendorff preparation upon mechanical stress (Lang *et al*. 1985), no effect is observed in intact animal upon marked volume expansion in the heart (Sakata *et al*. 1988), can be explained taking into account that the oxygen capacitance of a unit volume of blood is approx. 30 times higher than that of clear Krebs–Henseleit perfusion solution. All the physiological responses are caused by partial pressure of oxygen rather than the oxygen concentration (Dejours 1975). Because of the much lower oxygen capacitance of Krebs–Henseleit solution normally used in Langendorff preparations than of blood, the oxygen consumption of myocardial cells causes much greater reduction in cellular oxygen tension (and that of the fluid that has passed the heart) in Krebs–Henseleit solution than in blood. Consequently, the oxygen tension gradient in buffer‐perfused heart will be much greater as a result of either manipulation of the inflowing oxygen tension or mechanical stretch (increasing myocardial oxygen consumption) than in blood‐perfused heart. Notably, any increase in myocardial work can be related to increase in the oxygen consumption of the heart (Gutterman & Cowley 2006). The difference between Krebs–Henseleit solution and blood as perfusion media is seen in the fact that if red blood cells are added to buffers perfusing isolated hearts, both their mechanical and metabolic stability will be substantially improved (Beard *et al*. 2003, Schenkman *et al*. 2003, Moure *et al*. 2010, Kuzmiak‐Glancy *et al*. 2015). Further, the difference between the oxygen capacitance of blood and buffer explains why it is virtually impossible to elicit natriuretic peptide secretion by exposing healthy, conscious humans to hypoxic conditions at rest (Heinonen *et al*. 2014). Even though the oxygen saturation of inflowing blood decreases, the amount of oxygen delivered to the working cardiac muscle cells remains at least constant or may even increase, as hypoxia increases coronary flow (Feigl 1983). In blood‐perfused heart, if the oxygen tension of coronary artery remains above 35 mmHg, the oxygen tension of cardiac muscle cells does not decrease (Coburn *et al*. 1973). In the absence of blood cells, the amount of oxygen in the outflow from heart decreases already when the oxygen tension of the inflowing buffer is above atmospheric. This being the case, any increase in oxygen consumption of myocardial cells, as, for example, caused by mechanical stretch, will initially increase the partial pressure gradient of oxygen. As the oxygen sensing mechanisms usually sense oxygen tensions and their gradients, this situation will be seen as a marked effect of stretch on natriuretic peptide secretion.

On the basis of the above, one can predict that if coronary flow cannot increase as a response to either a decrease in oxygen tension in arterial blood or an increase in myocardial oxygen consumption, the natriuretic peptide levels will increase. If this prediction is right, one should see a change in BNP level especially after exercise (which decreases arterial oxygen saturation and increases myocardial oxygen consumption) even if systolic function is not disturbed. This has, indeed, been observed. The exercise‐induced release and synthesis of BNP and NT‐proBNP have been observed both in healthy individuals and in patients suffering from, for example, chronic heart failure (Pascual‐Figal *et al*. 2007, Maeder *et al*. 2011, Paraskevaidis *et al*. 2011, Woods *et al*. 2012). The reason behind the release of BNP during exercise is still somewhat unclear. It is probably not due to a reduction in arterial partial pressure (PO_2_) as the BNP level did not correlate with arterial PO_2_ (Zurek *et al*. 2013). However, the results are compatible with oxygen tension of myocardial cells being the trigger. It has been observed that intracellular O_2_ is reduced close to zero during exercise (e.g. Wagner 2012). Reduction in intracellular O_2_ activates hypoxia‐inducible factor (HIF) which among other things induces the production of BNP (Luo *et al*. 2006, Weidemann *et al*. 2008). Notably, also sleep apnoea activates HIF‐1*α* (e.g. review by Nanduri *et al*. 2015). Thus, although it is not unequivalently demonstrated, hypoxaemia during sleep apnoea could induce BNP release. Because of the possible role of intracellular oxygen tension in influencing BNP release, further studies measuring intracellular O_2_ levels are needed. It is also of some interest that systolic function can be preserved, and still the BNP level can increase in coronary disease (Palazzuoli *et al*. 2004); this would easily be compatible with oxygen tension (and its gradient) being the major signal for natriuretic peptide secretion, but harder to reconcile with mechanical stretch or pressure as such being the signal.

In conclusion, in the present study we have shown that the BNP level of perfusate is increased, when the oxygen tension of the inflowing buffer is decreased. If oxygen tension (and especially its gradient) is the major signal for natriuretic peptide secretion, the observed effects of both hypoxia and mechanical stretch can be reconciled, and the conclusion explains the different results obtained with isolated buffer‐perfused hearts, and blood‐perfused hearts in intact animals.

We would like to thank Dr. Olli Arjamaa for support and discussions about the study. The research was funded by Academy of Finland (project no. 258078).

## Conflict of interest

The authors declare no competing or financial interests.
